# Do Popular Physician-Rating Websites Provide Consistent Reviews for Academic and Community Hematology-Oncology Physicians?

**DOI:** 10.7759/cureus.112763

**Published:** 2026-07-16

**Authors:** Adam Zoubi, Bryan Coburn, John D Cambron, Kimberly Leeson, Alainya Tomanec, Peter Richman, Cameron Cooper, Charlotte Lochner, Cynthia Harris, Jeanelle Proctor, Giselle Murillo

**Affiliations:** 1 Internal Medicine, CHRISTUS Spohn Hospital, Texas A&amp;M University Naresh K. Vashisht College of Medicine, Corpus Christi, USA; 2 Emergency Medicine, CHRISTUS Spohn Hospital, Texas A&amp;M University Naresh K. Vashisht College of Medicine, Corpus Christi, USA; 3 Emergency Medicine, Baylor College of Medicine, CHRISTUS Children's, San Antonio, USA

**Keywords:** academic medicine, community oncology, hematology-oncology, online patient reviews, patient experience, physician rating websites, review volume

## Abstract

Background: Physician-rating websites are frequently used by patients and health systems seeking information about clinicians, but the reliability of these data for hematology-oncology physicians is unclear.

Objective: To evaluate review volume, rating scores, and physician-level associations on popular physician-rating websites for academic and community hematology-oncology physicians.

Methods: We conducted a retrospective cross-sectional analysis of 210 US hematology-oncology attending physicians, including 105 academic and 105 community/private physicians. Academic physicians were sampled from 21 academic cancer-center sampling blocks across five US regions, including four blocks each from the Northeast, Southeast, Midwest, and Southwest, and five from the West. Community/private physicians were sampled from nearby non-academic hospitals or oncology practices in the same geographic areas. Fellows, residents, advanced practice providers, non-attending physicians, and pediatric-only oncologists were excluded. Physician-rating data were collected from Healthgrades, Vitals, RateMDs, and WebMD. Continuous variables were summarized using means with standard deviations and medians with interquartile ranges. Academic and community physicians were compared using Mann-Whitney U tests for skewed continuous variables and chi-square tests for categorical variables. Associations between years in practice, review volume, and ratings were assessed using Spearman correlation. A blinded second reviewer abstracted a subset of physicians to assess interrater reliability.

Results: Among 210 hematology-oncology physicians, the mean total number of reviews across all four websites was 40.3 ± 99.7, with a median of 14.0 reviews (interquartile range (IQR): 4.0-38.8) and a range of 0-1030. Nine physicians (4.3%) had zero reviews, 63 (30.0%) had fewer than five reviews, and 83 (39.5%) had fewer than 10 reviews. Community/private physicians had higher review volume than academic physicians, with 56.0 ± 126.8 reviews vs. 24.6 ± 58.3 reviews, and median review counts of 24.0 (IQR: 5.0-55.0) vs. 10.0 (IQR: 3.0-25.0) (p = 0.0014). Academic physicians were more likely than community/private physicians to have fewer than 10 total reviews: 52/105 (49.5%) vs. 31/105 (29.5%) (p = 0.003). Among physicians with at least one available platform rating, the mean rating score was 4.39 ± 0.78 on a 1-5 scale. Mean rating scores were 4.51 ± 0.56 for community/private physicians and 4.28 ± 0.93 for academic physicians, but rating distributions did not differ significantly by cohort (p = 0.47). No meaningful gender-based differences were identified. Longer time in practice was associated with greater total review volume (Spearman's ρ = 0.379, p < 0.001) and lower mean rating scores (ρ = -0.255, p < 0.001). Interrater agreement was near-perfect across review presence, number of reviews, rating presence, average rating scores, and narrative comment presence, with kappa values ranging from 0.94 to 0.96.

Conclusion: Popular physician-rating websites offer limited, highly variable review volume for hematology-oncology physicians, with many clinicians receiving few or no reviews despite generally high average ratings. Community/private physicians had more reviews than academic physicians, while rating distributions were similarly high across cohorts. These results indicate that online physician ratings should be interpreted cautiously by patients, clinicians, and health systems.

## Introduction

Public physician-rating websites have become a prominent source of information for patients selecting clinicians. These sites commonly appear in internet search results and provide numerical scores, review counts, and narrative comments. National survey data show that many patients are aware of physician-rating sites and that a meaningful subset use them when choosing a physician; physicians also recognize that these public profiles can affect professional reputation and patient expectations [1,2].

The convenience of online ratings may support transparency, but it also allows a small and self-selected group of reviewers to shape a clinician's digital identity. A central interpretive problem is the denominator underlying a displayed score. Early studies found that many physicians had few or no reviews and that available reviews were predominantly favorable [3].

Analyses of commercial platforms have continued to show uneven profile coverage and small numbers of reviews for many clinicians [4]. Large longitudinal and cross-sectional studies have further demonstrated strong positive skew, with ratings clustered near the upper end of five-point scales [5,6].

When a physician has only one or a few reviews, a displayed average may be unstable, may change substantially after a single new review, and may not represent the broader patient population. Systematic reviews have identified multiple threats to the interpretability of physician-rating websites, including incomplete profile coverage, self-selection of reviewers, duplicate or outdated profiles, heterogeneous rating instruments, variable moderation policies, uncertain review authenticity, and platform-specific presentation [7-10].

These concerns do not mean that patient narratives lack value. Online comments may capture communication, access, courtesy, and care-coordination experiences that matter to patients. However, the credibility of a numerical rating depends on what it measures, how many observations support it, and whether the observations are current and representative. The relationship between public ratings and other measures of physician performance is inconsistent. Comparisons with peer-recognition lists, clinical quality indicators, value measures, and validated patient-experience surveys have reported absent, weak, or specialty-dependent associations [11-14].

In a large integrated health system, online physician scores were based on far fewer responses than internal patient surveys, and agreement between the two improved as the number of online reviews increased [14]. Recency also matters because a cumulative score can retain the influence of older reviews even when a physician's practice setting or performance has changed [15]. These findings support interpreting online ratings primarily as selected patient-experience signals rather than direct measures of technical quality or clinical outcomes. This issue may be particularly consequential in hematology-oncology. Care is commonly longitudinal, multidisciplinary, emotionally charged, and delivered during periods of substantial uncertainty. Patient experience may be affected by prognosis, treatment toxicity, disease progression, insurance barriers, scheduling, infusion-center operations, and communication across teams.

Some of these factors are clinically and personally important but do not map neatly onto the performance of one physician. Prior oncology-focused studies have described generally favorable online reviews of radiation oncologists on Healthgrades and Vitals [16,17], but adult hematologists and medical oncologists have not been systematically characterized across several major platforms while directly comparing academic and community/private practice settings.

The primary objective of this study was to quantify review volume and rating scores for US hematology-oncology physicians across Healthgrades, Vitals, RateMDs, and WebMD. Secondary objectives were to compare academic and community/private physicians, examine associations with physician characteristics, describe platform-specific availability, and assess the reproducibility of data abstraction. We hypothesized that many physicians would have low review volume, that community/private physicians would have more reviews than academic physicians, and that rating scores would show limited discrimination and minimal association with physician characteristics.

## Materials and methods

Study design, reporting, and ethics

We conducted a retrospective cross-sectional study of publicly available physician profiles and rating data. No intervention was performed, and the investigators did not contact physicians, patients, or website users. The manuscript was prepared with reference to the Strengthening the Reporting of Observational Studies in Epidemiology (STROBE) recommendations for cross-sectional studies [18].

Study protocol was submitted to CHRISTUS Health IRB, with submission Reference 016091, and on March 26, 2026, the study protocol was reviewed by the CHRISTUS Health IRB Chair and determined to meet the criteria for exempt category review under the provisions of 45 CFR 46.104 (a)(d) (approval period: March 26, 2026, to March 25, 2027).

Sampling frame and physician selection

The analytic sample consisted of 210 US-based attending physicians practicing adult hematology-oncology. Two equal cohorts were constructed: 105 physicians practicing in academic settings and 105 physicians practicing in community/private settings. Fellows, residents, other trainees, advanced practice providers, physicians in non-attending roles, and pediatric-only oncologists were excluded.

The fixed sample size followed the prespecified design of 21 geographic sampling blocks with five academic and five community/private physicians per block; no separate power calculation was used because the primary aim was descriptive. Academic sampling was organized around 21 cancer-center blocks distributed across five US regions: four blocks each in the Northeast, Southeast, Midwest, and Southwest, and five blocks in the West.

Academic centers were identified through the National Cancer Institute's cancer center directory. Five eligible physicians were selected from each academic block. For each block, a nearby nonacademic hospital or oncology practice was identified using the Centers for Medicare & Medicaid Services Care Compare resources, institutional websites, and fellowship program information. Sites listed as National Cancer Institute centers, directly affiliated academic sites, and institutions with a hematology-oncology fellowship program were excluded from the community/private cohort.

Five eligible community/private physicians were selected from each matched geographic area. The final sample was balanced by cohort within every region. From each selected center, five physicians were randomly selected per the eligibility rules mentioned above; when available, selections were divided to represent both male and female doctors. Selection was based on last name alphabetical order, to randomize the selection until each block's quota is met.

Data sources and abstraction

Reviewers abstracted data from Healthgrades, Vitals, RateMDs, and WebMD. For each physician and platform, the dataset recorded whether reviews were present, the displayed number of reviews, whether a numerical rating was available, the displayed average rating on a one-to-five scale, and whether narrative comments were present. Physician-level variables included practice cohort, US region, state, gender as coded from the source directories, degree type (MD or DO), and years in practice. Direct physician identifiers were used for profile matching and were replaced by study identification numbers for analysis.

Outcomes and data preparation

The primary outcome was total review volume per physician, defined as the sum of displayed review counts across all four websites. We also calculated the number of platforms with at least one review and summarized the proportions of physicians with 0 reviews, fewer than five reviews, fewer than 10 reviews, and fewer than 20 reviews. These thresholds were prespecified descriptive categories and were not treated as validated reliability cutoffs.

The principal rating outcome was the unweighted arithmetic mean of all available platform rating scores for each physician. Platforms without a displayed rating were omitted from that physician's mean. Physicians with no available rating on any platform were excluded from rating-score analyses but retained in all review-volume analyses. Platform-level review counts were analyzed with zeros included. The final analytic dataset contained complete values for cohort, region, gender, degree, and years in practice.

Range and logic checks were performed before analysis. One RateMDs rating-presence field was coded as 5 rather than a binary value and was recoded to 1 because an adjacent five-point rating and one review were recorded. One RateMDs narrative-comment field was recoded from 1 to 0 because both review presence and review count were 0. A WebMD rating was retained for one physician despite a 0 review count because rating availability and review volume were collected as separate platform fields. No review counts or rating scores were otherwise altered.

Interrater reliability

A blinded second reviewer independently repeated the abstraction for 25 physicians, as documented in the completed reliability workbook (15 academic and 10 community/private). Agreement for review presence, rating presence, and narrative-comment presence was evaluated across 100 physician platforms on the four different rating platforms.

Review-count agreement was summarized across the 100 platform-level count fields, and a two-way absolute agreement single-measure ICC was used for the physician-level total review count. Rating-score agreement was summarized across platform pairs for which both reviewers recorded a numerical score, using the same ICC model for physician-level mean rating [19]. Additionally, 95% confidence intervals were estimated using 5,000 bootstrap resamples, with physicians resampled as clusters for the platform-level categorical analyses.

## Results

Physician sample

The final sample included 210 physicians from 17 states: 105 academic and 105 community/private physicians. Regional representation was balanced by design, with 40 physicians each from the Northeast, Southeast, Midwest, and Southwest and 50 from the West. The sample included 114 physicians coded as male (54.3%) and 96 coded as female (45.7%). Gender distribution did not differ significantly by cohort (Pearson χ²(1) = 1.228; OR for male coding in community/private versus academic practice = 1.36, 95% CI: 0.79-2.34; p = 0.268). Overall, 201 physicians (95.7%) held an MD degree, and 9 (4.3%) held a DO degree; all nine DO physicians were in the community/private cohort (Fisher's exact test p = 0.003). Mean years in practice were 23.7 ± 11.6, and years in practice did not differ significantly between academic and community/private physicians (Mann-Whitney U = 6035.0, p = 0.235) (Table 1).

**Table 1:** Physician characteristics by practice setting N*t: number of total samples; N*a: number of academic samples; N*c: number of community samples; OR: odds ratio; N/A: not applicable ORs compare community/private physicians with academic physicians for the stated category. Pearson chi-square tests were used to assess gender, Fisher's exact test was used to assess degree type, and the Mann-Whitney U test was used to assess years in practice. The region was balanced by design.

Characteristic	N*t=210	N*a=105	N*c=105	Test statistic	Effect estimate (95% CI)	p-value
Gender coded as male	54.3%	50.5%	58.1%	Pearson χ²(1) = 1.228	OR = 1.36 (0.79-2.34)	0.268
Gender coded as female	45.7%	49.5%	41.9%	Pearson χ²(1) = 1.228	OR = 0.74 (0.43-1.27)	0.268
MD degree	95.7%	100.0%	91.4%	N/A	OR = 0.00 (0.00-0.48)	0.003
DO degree	4.3%	0.0%	8.6%	N/A	OR = infinity (2.08-infinity)	0.003
Years in practice mean	23.7	24.4	23.0	N/A	N/A	N/A
Years in practice median	20.0	23.0	20.0	Mann-Whitney U = 6035.0	N/A	0.235
Years in practice range	5-58	5-58	5-57	N/A	N/A	N/A
Northeast	40	20	20	N/A	N/A	N/A
Southeast	40	20	20	N/A	N/A	N/A
Midwest	40	20	20	N/A	N/A	N/A
Southwest	40	20	20	N/A	N/A	N/A
West	50	25	25	N/A	N/A	N/A

Aggregate review volume

Across the four websites, physicians had a mean of 40.3 ± 99.7 total reviews and a median of 14.0 (IQR: 4.0-38.8), with a range of 0-1030. The large difference between the mean and median reflected a highly right-skewed distribution. Nine physicians (4.3%) had no reviews on any platform; 63 (30.0%) had fewer than five reviews; 83 (39.5%) had fewer than 10 reviews; and 120 (57.1%) had fewer than 20 reviews. Reviews were present on all four platforms for only 27 physicians (12.9%); 50 physicians (23.8%) had reviews on zero or one platform. Community/private physicians had significantly more reviews than academic physicians (mean: 56.0 ± 126.8 vs 24.6 ± 58.3; median: 24.0 (IQR: 5.0-55.0) vs 10.0 (IQR: 3.0-25.0); p = 0.001). Academic physicians were more likely to have fewer than 10 total reviews (49.5% vs 29.5%; p = 0.003) and fewer than 20 reviews (67.6% vs 46.7%; p=0.002). The proportions with no reviews did not differ significantly (4.8% vs 3.8%; p = 0.733) (Table 2).

**Table 2:** Aggregate review-volume and rating outcomes by practice setting N*t: number of total samples; N*a: number of academic samples; N*c: number of community samples; N/A: not applicable Counts below review thresholds are cumulative. Rating summaries include only physicians with at least one available numerical rating. Mann-Whitney U tests were used to compare continuous distributions, and Pearson chi-square tests were used to compare categorical outcomes.

Outcome	N*t=210	N*a=105	N*c=105	Test statistic	Effect estimate (95% CI)	p-value
Total reviews mean	40.3	24.6	56.0	N/A	N/A	N/A
Total reviews median	14.0	10.0	24.0	Mann-Whitney U = 4105.0	N/A	0.0014
Total reviews range	0-1030	0-511	0-1030	N/A	N/A	N/A
No reviews	4.3%	4.8%	3.8%	Pearson χ²(1) = 0.116	OR = 0.79 (0.21-3.04)	0.733
Fewer than five reviews	30.0%	35.2%	24.8%	Pearson χ²(1) = 2.744	OR = 0.60 (0.33-1.10)	0.098
Fewer than 10 reviews	39.5%	49.5%	29.5%	Pearson χ²(1) = 8.786	OR = 0.43 (0.24-0.75)	0.003
Fewer than 20 reviews	57.1%	67.6%	46.7%	Pearson χ²(1) = 9.411	OR = 0.42 (0.24-0.73)	0.002
At least one rating	95.7%	95.2%	96.2%	Pearson χ²(1) = 0.116	OR = 1.26 (0.33-4.84)	0.733
Mean rating mean	4.39	4.28	4.51	N/A	N/A	0.467
Mean rating median	4.60	4.57	4.60	Mann-Whitney U = 4753.5	N/A	N/A
Mean rating exactly 5.0	29.4%	33.0%	25.7%	N/A	N/A	N/A
Mean rating >=4.0	79.1%	73.0%	85.1%	N/A	N/A	N/A
Any narrative comment	84.3%	80.0%	88.6%	Pearson χ²(1) = 2.912	OR = 1.94 (0.90-4.18)	0.088

Rating scores

At least one numerical platform rating was available for 201 physicians (95.7%). Among rated physicians, the mean physician level rating was 4.39 ± 0.78, and the median was 4.60 (IQR: 4.07-5.00). Fifty-nine rated physicians (29.4%) had a physician-level mean of exactly 5.0, and 159 (79.1%) had a mean rating of at least 4.0. Mean ratings were 4.51 ± 0.56 for community/private physicians and 4.28 ± 0.93 for academic physicians, but the distributions did not differ significantly (p = 0.467). Rating availability and the presence of any narrative comment also did not differ significantly by cohort. Review volume and rating distributions did not differ meaningfully by gender (p = 0.114 and p = 0.664, respectively).

Platform-specific findings

Coverage differed substantially by website. Healthgrades had the greatest review availability (175/210, 83.3%), followed by WebMD (158/210, 75.2%) and Vitals (153/210, 72.9%); RateMDs had reviews for only 29 physicians (13.8%). Median review counts, including zeros, were 5.0 on Healthgrades, 4.0 on WebMD, 3.0 on Vitals, and 0 on RateMDs. Community/private physicians had greater Healthgrades review availability (88.6% vs 78.1%; p = 0.042) and higher Healthgrades review counts (median: 8 vs 3; p < 0.001). Other platform-specific review-volume comparisons were not statistically significant at the 0.05 level (Table 3).

**Table 3:** Platform-specific review availability and review volume N*t: number of total samples; N*a: number of academic samples; N*c: number of community samples; N/A: not applicable Review-count summaries include physicians with zero reviews. Availability was compared using Pearson chi-square tests, and review-count distributions were compared using Mann-Whitney U tests.

Platform	Measure	N*t=210	N*a=105	N*c=105	Test statistic	Effect estimate (95% CI)	p-value
Healthgrades	Any review	83.3%	78.1%	88.6%	Pearson χ²(1) = 4.149	OR = 2.17 (1.02-4.64)	0.042
Healthgrades	Review count mean	14.9	7.7	22.1	N/A	N/A	N/A
Healthgrades	Review count median	5.0	3.0	8.0	Mann-Whitney U = 3597.5	N/A	<0.001
Vitals	Any review	72.9%	72.4%	73.3%	Pearson χ²(1) = 0.024	OR = 1.05 (0.57-1.93)	0.877
Vitals	Review count mean	12.4	8.4	16.4	N/A	N/A	N/A
Vitals	Review count median	3.0	3.0	5.0	Mann-Whitney U = 4768.0	N/A	0.088
RateMDs	Any review	13.8%	11.4%	16.2%	Pearson χ²(1) = 1.000	OR = 1.50 (0.68-3.31)	0.317
RateMDs	Review count mean	0.6	0.4	0.8	N/A	N/A	N/A
RateMDs	Review count median	0.0	0.0	0.0	Mann-Whitney U = 5242.0	N/A	0.306
WebMD	Any review	75.2%	75.2%	75.2%	Pearson χ²(1) = 0.000	OR = 1.00 (0.53-1.87)	1
WebMD	Review count mean	12.4	8.1	16.7	N/A	N/A	N/A
WebMD	Review count median	4.0	3.0	6.0	Mann-Whitney U = 4688.0	N/A	0.059

Platform rating means were uniformly high, ranging from 4.29 on Vitals to 4.46 on WebMD. No platform showed a statistically significant difference in rating-score distribution between academic and community/private physicians. Narrative comments were most commonly present on Healthgrades (70.0%) and least commonly present on RateMDs (13.8%). Healthgrades narrative comments were more common among community/private physicians (77.1% vs 62.9%; p=0.024); this and other platform-specific findings should be interpreted as exploratory, as no multiplicity adjustment was applied (Table 4).

**Table 4:** Platform-specific rating availability, rating score, and narrative-comment presence N/A: not applicable Rating availability and narrative-comment presence were compared using Pearson chi-square tests, and rating distributions were compared using Mann-Whitney U tests.

Platform	Measure	Overall	Academic	Community/private	Test statistic	Effect estimate (95% CI)	p-value
Healthgrades	Rating available	83.3%	78.1%	88.6%	Pearson χ²(1) = 4.149	OR = 2.17 (1.02-4.64)	0.042
Healthgrades	Rating score mean	4.34	4.24	4.43	N/A	N/A	N/A
Healthgrades	Rating score median	4.70	4.80	4.60	Mann-Whitney U = 3867.0	N/A	0.869
Healthgrades	Narrative comment	70.0%	62.9%	77.1%	Pearson χ²(1) = 5.102	OR = 1.99 (1.09-3.65)	0.024
Vitals	Rating available	72.9%	72.4%	73.3%	Pearson χ²(1) = 0.024	OR = 1.05 (0.57-1.93)	0.877
Vitals	Rating score mean	4.29	4.16	4.41	N/A	N/A	N/A
Vitals	Rating score median	4.50	4.25	4.50	Mann-Whitney U = 2520.5	N/A	0.133
Vitals	Narrative comment	61.4%	59.0%	63.8%	Pearson χ²(1) = 0.502	OR = 1.22 (0.70-2.13)	0.478
RateMDs	Rating available	13.8%	11.4%	16.2%	Pearson χ²(1) = 1.000	OR = 1.50 (0.68-3.31)	0.317
RateMDs	Rating score mean	4.46	4.81	4.21	N/A	N/A	N/A
RateMDs	Rating score median	4.88	5.00	4.66	Mann-Whitney U = 142.0	N/A	0.063
RateMDs	Narrative comment	13.8%	11.4%	16.2%	Pearson χ²(1) = 1.000	OR = 1.50 (0.68-3.31)	0.317
WebMD	Rating available	75.7%	76.2%	75.2%	Pearson χ²(1) = 0.026	OR = 0.95 (0.51-1.78)	0.872
WebMD	Rating score mean	4.46	4.39	4.53	N/A	N/A	N/A
WebMD	Rating score median	5.00	5.00	5.00	Mann-Whitney U = 3047.5	N/A	0.677
WebMD	Narrative comment	39.5%	33.3%	45.7%	Pearson χ²(1) = 3.367	OR = 1.68 (0.96-2.94)	0.067

Physician-level associations

Longer time in practice was associated with greater total review volume (Spearman ρ = 0.379; p < 0.001) and with lower mean rating scores (ρ = -0.255; p < 0.001). Total review volume was also inversely associated with the mean rating (ρ = -0.362; p < 0.001), consistent with a greater opportunity for ratings to move away from the upper boundary as the number of observations increased. These associations were modest and do not establish causation (Table 5).

**Table 5:** Spearman correlations among years in practice, review volume, and mean rating Rating analyses included physicians with at least one available rating. Correlations were unadjusted and exploratory.

Association	n	Spearman ρ	p-value
Years in practice vs total review volume	210	0.379	<0.001
Years in practice vs mean rating	201	-0.255	<0.001
Total review volume vs mean rating	201	-0.362	<0.001

Interrater reliability

The blinded reliability sample included 25 physicians (15 academic and 10 community/private), yielding 100 physician-platform comparisons across the four separate platforms. Exact agreement was 97% for review presence, 98% for rating presence, and 97% for narrative-comment presence; corresponding kappa values ranged from 0.94 to 0.96. Platform-level review counts matched exactly in 96% of comparisons, and the intraclass correlation coefficient (ICC) for physician-level total review count was 0.958. Numerical rating scores matched exactly in all 60 platform pairs in which both reviewers recorded a score. The ICC for physician-level mean rating among 23 physicians rated by both reviewers was 0.9996 (Table 6).

**Table 6:** Blinded interrater reliability for data abstraction CI: confidence interval; ICC(A,1): two-way absolute-agreement, single-measure intraclass correlation coefficient; N/A: not applicable Bootstrap confidence intervals were estimated using 5,000 resamples. The reliability sample completed in the workbook was n = 25.

Field	Analysis level	n	Exact agreement	Coefficient	Estimate	95% CI	Notes
Review presence	Physician-platform	100	97/100 (97.0%)	Cohen kappa	0.937	0.858-1.000	Binary field
Rating presence	Physician-platform	100	98/100 (98.0%)	Cohen kappa	0.958	0.888-1.000	Binary field
Narrative-comment presence	Physician-platform	100	97/100 (97.0%)	Cohen kappa	0.94	0.873-1.000	Binary field
Review count	Platform field	100	96/100 (96.0%)	N/A	N/A	N/A	Exact count agreement
Total review count	Physician total	25	N/A	ICC(A,1)	0.958	0.951-1.000	Absolute-agreement single measure
Rating score	Shared platform rating	60	60/60 (100.0%)	N/A	N/A	N/A	Exact score agreement
Mean rating score	Physician mean	23	N/A	ICC(A,1)	0.9996	0.997-1.000	Absolute-agreement single measure

## Discussion

Principal findings

In this geographically structured sample of 210 US hematology-oncology physicians, online review volume was sparse for many clinicians and extremely variable across physicians and platforms. Four of the 10 physicians had fewer than 10 total reviews across four websites, more than half had fewer than 20, and only 12.9% had reviews on every platform. Community/private physicians accumulated substantially more reviews than academic physicians, yet rating-score distributions were similarly high in both cohorts.

Nearly all physicians had at least one displayed rating, but the physician-level mean clustered near the top of the five-point scale. Platform coverage varied sharply, from 83.3% on Healthgrades to 13.8% on RateMDs. The results support the study hypothesis that review volume is often limited and that high rating scores provide relatively little discrimination among hematology-oncology physicians.

Sparse review volume and the denominator problem

The review count underlying a displayed score is essential context. A physician with one five-star review and a physician with 100 reviews averaging five stars may appear identical in a search result, although the stability and representativeness of those scores are very different. Prior research has repeatedly documented sparse and uneven review volume [3-6], and a large integrated-system comparison found that public ratings were based on far fewer responses than internal patient surveys; their correlation with internal scores improved as online review volume increased [14]. The present study expands this concern to adult hematology-oncology across four platforms. The fewer-than-five, fewer-than-10, and fewer-than-20 categories should not be interpreted as validated thresholds. Rather, they show how often patients may encounter scores supported by relatively small denominators.

The distribution was also highly right-skewed: the median physician had 14 reviews, whereas the mean was 40 because a small number of physicians had several hundred reviews. This heterogeneity means that a single overall average obscures substantial differences in the amount of information available for individual clinicians. It similarly complicates comparisons between physicians and health systems because profiles with larger review counts may reflect higher patient volume, longer online exposure, active review solicitation, or platform-specific aggregation rather than better or worse care.

Academic-community differences

Community/private physicians had a median of 24 reviews, compared with 10 for academic physicians, and the academic cohort was more likely to have fewer than 10 or 20 reviews. The study was not designed to identify the mechanism for this difference. Possible explanations include differences in ambulatory encounter volume, continuity of care, patient solicitation practices, marketing or reputation-management activities, and the extent to which patients seek out an individual physician rather than an academic cancer center brand.

Academic oncologists may also care for more tertiary referrals, highly subspecialized conditions, or patients whose experience is distributed across large multidisciplinary teams. These explanations are hypotheses only because the dataset did not include patient volume, referral patterns, clinic structure, or review-solicitation practices.

High scores, ceiling effects, and physician characteristics

Despite sparse review volume, displayed ratings were generally favorable: 79.1% of rated physicians had a mean rating of at least 4.0, and 29.4% had a mean of exactly 5.0. This positive skew is consistent with prior studies of online physician ratings [5,6]. A ceiling effect limits discrimination because physicians with different experiences and outcomes may receive similarly high scores, while a small number of negative reviews can excessively affect profiles with few observations. The absence of a significant academic-community difference in rating distributions, despite a clear difference in review volume, suggests that review count and rating score capture different features of the online profile.

Longer time in practice was associated with more reviews, which is plausible because physicians with longer careers have had more opportunities to accumulate an online footprint. Years in practice were also modestly associated with lower mean ratings, and review volume itself was inversely associated with rating. The results should be interpreted cautiously. Ratings may reflect cohort effects, changes in platform use, practice transitions, or a mathematical tendency for averages based on more observations to move away from a perfect score. Review recency was unavailable, and prior work shows that older reviews can continue to influence cumulative scores [15]. No meaningful gender-based differences in review volume or ratings were identified.

Platform heterogeneity

The same physician can have a significantly different digital footprint depending on the website a patient visits. Healthgrades, Vitals, and WebMD each contained reviews for most sampled physicians, whereas RateMDs contained reviews for fewer than one in seven. Community/private physicians had greater Healthgrades coverage and review volume, but rating score distributions did not differ by cohort on any platform. These findings reinforce the idea that a physician's online reputation is not a single, stable construct. Platform-specific search prominence, profile creation, data partnerships, moderation rules, and review solicitation may affect both coverage and score presentation [7-10]. Patients who consult only one website may therefore receive an incomplete picture.

Interpretation in oncology and relation to quality

Online reviews may provide useful information about communication, empathy, access, staff interactions, and care coordination. These dimensions matter in oncology, where difficult conversations and long-term relationships are common. However, numerical ratings should not be equated with technical quality or cancer outcomes. Prior studies comparing public ratings with peer review, quality, value, or validated patient-experience measures have found mixed or weak associations [11-14]. Oncology ratings may also be influenced by disease trajectory, toxicity, prognosis, insurance barriers, waiting time, and institutional processes beyond the physician's control. The present study measured availability and distribution, not criterion validity, and therefore cannot determine whether high or low ratings accurately reflect the quality of care.

Reliability and implications

The duplicate-abstraction analysis showed excellent reproducibility of the study's data-collection process. This supports the internal reliability of locating profiles and transcribing displayed fields, although discrepancies occurred when one reviewer located an additional profile or review set. Importantly, abstraction reliability is not the same as the reliability or validity of patient-generated ratings themselves. High kappa and ICC values indicate that reviewers could reproduce the website data; they do not establish that those data are representative, current, or clinically valid.

Patients should consider the number and recency of reviews, along with the displayed score, and use online ratings as one source among several. Platforms should improve understandability by displaying denominators and review dates prominently, identifying when profiles combine data from several sources, explaining moderation and profile-matching practices, and communicating uncertainty when counts are small. Health systems can complement commercial ratings with standardized patient-experience data collected systematically from larger numbers of encounters. Subsequent studies should examine review recency, narrative themes, patient volume, case mix, review solicitation, longitudinal change, and associations with standardized experience and clinical-quality measures. Sensitivity analyses using review-weighted rather than platform-unweighted physician ratings would also be informative.

Strengths and limitations

Study strengths include equal academic and community/private cohorts, geographic coverage across five US regions, inclusion of physicians with no reviews, assessment of four major websites, physician-level aggregation, and blinded duplicate abstraction. The design directly evaluated the denominator underlying online scores and distinguished review availability from rating availability. Logic checks and transparent recoding of two inconsistent fields further reduced avoidable data-entry errors.

Several limitations should be considered. First, this was a cross-sectional snapshot of dynamic websites; counts, scores, algorithms, profile availability, and search prominence can change over time. Second, the structured regional sample was not a probability sample of all US hematology-oncology physicians, and the exact roster-selection procedure must be reported prior to submission. Third, public profile matching can be affected by duplicate profiles, name changes, multiple locations, and outdated specialty information. Fourth, online reviewers are self-selected and cannot be verified from public data, and summed counts across platforms may include the same patient more than once. Fifth, the physician-level rating was an unweighted mean of available platform scores, even though platforms had different review counts. Finally, the study did not measure review recency, narrative content, patient volume, case mix, clinical outcomes, standardized patient experience scores, or review solicitation practices.

## Conclusions

Across four public physician-rating websites, US hematology-oncology physicians had highly variable review volume, and a substantial proportion had few or no reviews despite generally high average ratings. Community/private physicians accumulated more reviews than academic physicians, but rating distributions were similarly concentrated near the upper end of the scale. Platform coverage differed substantially. Online ratings should be interpreted cautiously by patients, clinicians, and health systems. With explicit attention to review count, platform coverage, and recency, it should not be used as an indicator of clinical quality alone.

## References

[1] Hanauer DA, Zheng K, Singer DC, Gebremariam A, Davis MM (2014). Public awareness, perception, and use of online physician rating sites. JAMA.

[2] Holliday AM, Kachalia A, Meyer GS, Sequist TD (2017). Physician and patient views on public physician rating websites: a cross-sectional study. J Gen Intern Med.

[3] Lagu T, Hannon NS, Rothberg MB, Lindenauer PK (2010). Patients' evaluations of health care providers in the era of social networking: an analysis of physician-rating websites. J Gen Intern Med.

[4] Lagu T, Metayer K, Moran M, Ortiz L, Priya A, Goff SL, Lindenauer PK (2017). Website characteristics and physician reviews on commercial physician-rating websites. JAMA.

[5] Kadry B, Chu LF, Kadry B, Gammas D, Macario A (2011). Analysis of 4999 online physician ratings indicates that most patients give physicians a favorable rating. J Med Internet Res.

[6] Gao GG, McCullough JS, Agarwal R, Jha AK (2012). A changing landscape of physician quality reporting: analysis of patients' online ratings of their physicians over a 5-year period. J Med Internet Res.

[7] Emmert M, Sander U, Pisch F (2013). Eight questions about physician-rating websites: a systematic review. J Med Internet Res.

[8] Hong YA, Liang C, Radcliff TA, Wigfall LT, Street RL (2019). What do patients say about doctors online? A systematic review of studies on patient online reviews. J Med Internet Res.

[9] Mulgund P, Sharman R, Anand P, Shekhar S, Karadi P (2020). Data quality issues with physician-rating websites: systematic review. J Med Internet Res.

[10] Guetz B, Bidmon S (2023). The credibility of physician rating websites: a systematic literature review. Health Policy.

[11] McGrath RJ, Priestley JL, Zhou Y, Culligan PJ (2018). The validity of online patient ratings of physicians: analysis of physician peer reviews and patient ratings. Interact J Med Res.

[12] Daskivich TJ, Houman J, Fuller G, Black JT, Kim HL, Spiegel B (2018). Online physician ratings fail to predict actual performance on measures of quality, value, and peer review. J Am Med Inform Assoc.

[13] Chen J, Presson A, Zhang C, Ray D, Finlayson S, Glasgow R (2018). Online physician review websites poorly correlate to a validated metric of patient satisfaction. J Surg Res.

[14] Okike K, Uhr NR, Shin SY, Xie KC, Kim CY, Funahashi TT, Kanter MH (2019). A comparison of online physician ratings and internal patient-submitted ratings from a large healthcare system. J Gen Intern Med.

[15] Wang W, Luo J, Dugas M, Gao GG, Agarwal R, Werner RM (2022). Recency of online physician ratings. JAMA Intern Med.

[16] Prabhu AV, Randhawa S, Clump D, Heron DE, Beriwal S (2018). What do patients think about their radiation oncologists? An assessment of online patient reviews on Healthgrades. Cureus.

[17] Randhawa S, Viqar A, Strother J, Prabhu AV, Xia F, Heron D, Beriwal S (2018). How do patients rate their radiation oncologists in the modern era: an analysis of Vitals.com. Cureus.

[18] von Elm E, Altman DG, Egger M, Pocock SJ, Gotzsche PC, Vandenbroucke JP (2007). The Strengthening the Reporting of Observational Studies in Epidemiology statement: guidelines for reporting observational studies. Lancet.

[19] Koo TK, Li MY (2016). A guideline of selecting and reporting intraclass correlation coefficients for reliability research. J Chiropr Med.

